# Evaluation of Cross-Immunogenicity of Ferret Antisera Following Immunization with H5N1 Vaccine Strains

**DOI:** 10.3390/vaccines14040301

**Published:** 2026-03-27

**Authors:** Seungyeon Kim, Eun Young Jang, Seo Young Moon, Eun Bee Choi, Hye Won Lee, Min-Suk Song, Beom Kyu Kim, YooKyoung Lee, In-Ohk Ouh

**Affiliations:** 1 Division of Vaccine Development Coordination, Center for Vaccine Research, National Institute of Infectious Diseases, National Institute of Health, Korea Disease Control and Prevention Agency, Cheongju 28160, Republic of Korea; hatmddus135@korea.kr (S.K.); sky11kk@korea.kr (E.Y.J.); msy1477@korea.kr (S.Y.M.); dmsql2274@korea.kr (E.B.C.); hwlee45@korea.kr (H.W.L.); leeykyoung@korea.kr (Y.L.); 2 College of Veterinary Medicine, Institute for Veterinary Biomedical Science, Kyungpook National University, Daegu 41566, Republic of Korea; 3 Department of Microbiology, Chungbuk National University Medical School, Cheongju 28160, Republic of Korea; songminsuk@chungbuk.ac.kr (M.-S.S.); rick960426@naver.com (B.K.K.)

**Keywords:** avian influenza, candidate vaccine virus, cross-neutralization, One Health

## Abstract

Background: Highly pathogenic avian influenza H5N1 viruses of clade 2.3.4.4b have spread globally since 2021, causing extensive outbreaks in avian populations and repeated spillovers into diverse mammalian hosts, including humans. These cross-species transmission events highlight ongoing pandemic risks and underscore the need for vaccine strategies that reflect viral evolution at the human–animal interface. Despite the availability of licensed H5 vaccines and newly recommended World Health Organization (WHO) candidate vaccine viruses (CVVs), the extent to which these vaccines elicit cross-reactive antibody responses against contemporary clade 2.3.4.4b viruses, including mammalian spillover isolates of avian origin, remains incompletely characterized. Method: In this study, ferret antisera were generated using four WHO-recommended H5 CVVs, including a clade 1 strain (A/Vietnam/1194/2004) and three clade 2.3.4.4b strains (A/Astrakhan/3212/2020, A/American wigeon/South Carolina/22-000345-001/2021, and A/Ezo red fox/Hokkaido/1/2022), formulated with alum adjuvant to reflect licensed vaccine formulation used in national preparedness programs. Antibody responses and cross-reactive activity were evaluated using hemagglutination inhibition (HI) and microneutralization (MN) assays against homologous vaccine strains and a feline-origin clade 2.3.4.4b H5N1 field isolate from Korea, A/Feline/Korea/SNU-01/2023. Results: Antisera induced by clade 2.3.4.4b CVVs showed cross-reactive antibody responses against homologous and heterologous clade 2.3.4.4b viruses and demonstrated measurable HI and MN responses against the feline-origin field isolate. In contrast, antisera raised against the clade 1 Vietnam CVV exhibited limited cross-reactivity against clade 2.3.4.4b viruses. Overall, clade 2.3.4.4b CVVs generally showed higher antibody responses than the clade 1 vaccine strain across multiple panels. Conclusions: These findings provide descriptive insights into antigenic differences between clade 1 and clade 2.3.4.4b viruses and support the antigenic relevance of clade 2.3.4.4b CVVs for contemporary H5N1 strains. This study highlights the importance of ongoing antigenic evaluation to inform vaccine strain selection within a One Health framework.

## 1. Introduction

Highly pathogenic avian influenza (HPAI) viruses continue to pose a substantial global threat to animal and public health, with H5N1 viruses of particular concern due to their expanding host range and increasing frequency of spillover into mammalian hosts. Since late 2021, clade 2.3.4.4b H5N1 viruses have spread globally, causing widespread outbreaks in poultry and wild birds and repeated transmission events in terrestrial and marine mammals [1,2,3,4,5]. These cross-species transmissions highlight the evolving ecological and epidemiological dynamics of HPAI viruses and raise concerns about their zoonotic and pandemic potential.

In addition to widespread animal infections, severe human infections caused by clade 2.3.4.4b H5N1 viruses have been reported in South America, including Ecuador and Chile [3,4]. In 2024, spillover of HPAI A (H5N1) into dairy cattle was confirmed across multiple herds in the United States, accompanied by sporadic human infections associated with direct animal contact or exposure to contaminated raw milk [6,7,8]. These events have heightened concerns regarding mammal-to-mammal transmission and increased zoonotic risk at the human–animal interface.

Similar cross-species transmission events have been reported in Europe and Asia. During a 2020 outbreak in Astrakhan, Russia, a clade 2.3.4.4b H5N8 virus was isolated from poultry workers, representing one of the earliest confirmed human infections associated with this lineage [9,10]. Although classified as H5N8, the hemagglutinin of A/Astrakhan/3212/2020 showed close genetic and antigenic similarity to contemporary clade 2.3.4.4b H5N1 viruses. Consequently, this strain was selected as a World Health Organization (WHO)-recommended candidate vaccine virus (CVV). Subsequent antigenic analyses demonstrated that ferret antisera raised against this CVV could recognize and neutralize recent human H5N1 isolates, including A/Texas/37/2024, indicating broad cross-reactivity within clade 2.3.4.4b viruses [7,8,11]. Accordingly, Astrakhan-derived H5 vaccines formulated with oil-in-water adjuvants such as MF59 or AS03 are currently under clinical evaluation in adults (e.g., NCT05874713 for MF59 formulation; NCT05975840 for AS03- adjuvanted formulation) [12,13].

The Republic of Korea has experienced similar patterns of HPAI virus circulation. Clade 2.3.4.4b H5N1 viruses were first detected in wild mandarin ducks in October 2021 (A/mandarin duck/Korea/WA585/2021), followed by widespread outbreaks in poultry farms and continued detection in wild birds [14,15]. Notably, avian-origin H5N1 viruses have also spilled over into mammals in Korea. In July 2023, mass infections and fatalities were reported among shelter cats in Seoul, representing one of the first large-scale mammalian outbreaks in the country [16]. More recently, in March 2025, a clade 2.3.4.4b H5N1 virus was isolated from a wild leopard cat in Jeollanam-do, marking the first confirmed fatal HPAI infection in a wild mammal in Korea [17]. These findings indicate that HPAI viruses in Korea are no longer confined to avian hosts and increasingly involve mammalian species.

Despite ongoing viral evolution, currently licensed H5N1 vaccines in Korea and other countries are based on earlier strains, including clade 1 (A/Vietnam/1194/2004) and clade 2.1 (A/Indonesia/2005). Although these adjuvanted vaccines can induce cross-neutralizing antibodies against clade 2.3.4.4b viruses, the magnitude and breadth of responses vary and may not fully extend to mammalian-derived spillover isolates [18]. In response to the global spread of clade 2.3.4.4b H5N1 viruses, the WHO has recommended three clade 2.3.4.4b CVVs—A/Astrakhan/3212/2020, A/American wigeon/South Carolina/22-000345-001/2021, and A/Ezo red fox/Hokkaido/1/2022—for pandemic preparedness [19,20]. However, the extent of cross-reactivity between licensed clade 1 vaccines and these newly recommended CVVs, particularly against mammalian spillover isolates, remains incompletely characterized [18,21].

In this study, we generated ferret antisera using WHO-recommended CVVs from clade 1 and clade 2.3.4.4b, formulated with alum adjuvant to reflect licensed H5 vaccine formulations. We evaluated immunogenicity and cross-neutralizing activity using hemagglutination inhibition (HI) and microneutralization (MN) assays against vaccine strains, newly recommended CVVs, and a feline-origin clade 2.3.4.4b H5N1 isolate representing avian-to-mammalian spillover. These findings provide immunological evidence to inform vaccine strain selection and support pandemic preparedness strategies within a One Health framework.

## 2. Materials and Methods

### 2.1. Vaccine Strains and Viruses

WHO-recommended H5 CVVs were obtained from the following sources: A/Vietnam/1194/2004 (H5N1, Clade 1) from GC Pharma, Yongin, Republic of Korea; A/Astrakhan/3212/2020 (H5N8, Clade 2.3.4.4b) from the Center for Biologics Evaluation and Research (CBER), U.S. Food and Drug Administration (FDA), Silver Spring, MD, USA; A/American wigeon/South Carolina/22-000345-001/2021 (H5N1, Clade 2.3.4.4b) from the Centers for Disease Control and Prevention (CDC), Atlanta, GA, USA; and A/Ezo red fox/Hokkaido/1/2022 (H5N1, Clade 2.3.4.4b) from the National Institute of Infectious Diseases (NIID), Tokyo, Japan. In addition, A/Feline/Korea/SNU-01/2023 (H5N1, Clade 2.3.4.4b), which was isolated during a mass mortality event at a cat shelter in Yongsan, Seoul, in July 2023, was included for cross-neutralization analyses. All CVVs used in this study were generated by reverse genetics and contain modified hemagglutinin (HA) genes in which the polybasic cleavage site has been removed. As a result, these viruses are attenuated and classified as low-pathogenic avian influenza viruses, enabling safe propagation in embryonated chicken eggs under standard laboratory conditions. The CVVs were propagated in 10-day-old specific pathogen-free (SPF) embryonated chicken eggs under standard conditions for influenza virus amplification.

### 2.2. Ferret Immunization and Antisera Collection

**Figure 1:**
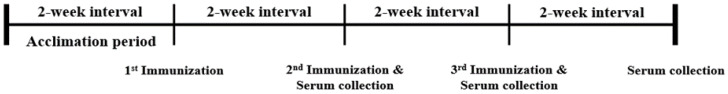
Immunization schedule and serum collection from ferrets. Ferrets were immunized intramuscularly with inactivated H5 CVVs formulated with alum adjuvant at weeks 0, 2, and 4. Sera were collected two weeks after the final immunization (week 6) and used for subsequent HI and MN assays.

Six-week-old female ferrets (Inscience, Daejeon, Republic of Korea) were anesthetized and immunized intramuscularly with 25 µg of each inactivated CVV—A/Vietnam/1194/2004, A/Astrakhan/3212/2020, A/American wigeon/South Carolina/22-000345-001/2021, and A/Ezo red fox/Hokkaido/1/2022—formulated with Alum adjuvant (Sigma-Aldrich Co., St. Louis, MO, USA). Antigens were mixed with alum at a 1:1 (*v*/*v*) ratio prior to administration. Due to limited animal availability, one ferret was immunized with the clade 1 Vietnam CVV, whereas two ferrets each were used for each clade 2.3.4.4b CVV for a total of seven animals. All ferrets were vaccinated three times at two-week intervals, and sera were collected two weeks after the final immunization. The three-dose immunization schedule was selected to enhance antibody responses and enable evaluation of cross-reactive immunity across antigenically distinct H5 viruses. Sera from each animal were tested in technical replicate as described below (Figure 1). Although sera were also collected after the first and second immunizations, the available volumes were insufficient to perform both HI and MN assays in a consistent and technically reliable manner across all groups; therefore, only sera collected after the final immunization were used for all subsequent analyses.

All animal procedures were conducted in accordance with the guidelines of the Institutional Animal Care and Use Committee (IACUC) of Chungbuk National University, and the study was approved under protocol number CBU-IACUC-24-0044-01.

### 2.3. H5N1 Neutralization Determination by Hemagglutination Inhibition (HI) Assay

HI assays were performed using a 1% suspension of chicken red blood cells. CVVs (A/Vietnam/1194/2004, A/Astrakhan/3212/2020, A/American wigeon/South Carolina/22-000345-001/2021, and A/Ezo red fox/Hokkaido/1/2022), which had been propagated in 10-day-old embryonated chicken eggs, were handled under biosafety level 2 (BSL-2) conditions, whereas assays with HPAI virus A/Feline/Korea/SNU-01/2023 were conducted in enhanced BSL-3 facilities. Serum samples were pretreated with receptor-destroying enzyme (RDE) and subsequently inactivated with heat. RDE-treated sera were incubated with four hemagglutinating units of a virus for 30 min at room temperature. The HI endpoint titer was determined as the highest serum dilution that resulted in complete inhibition of hemagglutination. The HI assay detection limit was 1:10, and titers below this value were assigned a value of 10 for graphical presentation and statistical analysis. A titer of ≥40 was considered indicative of seropositivity. Each sample was assayed in two independent HI experiments.

### 2.4. H5N1 Antibody Titer Determination by Microneutralization (MN) Assay

MN assays were performed using Madin–Darby canine kidney (MDCK) cells as the host system. Antigens included WHO-recommended CVVs (A/Vietnam/1194/2004, A/Astrakhan/3212/2020, A/American wigeon/South Carolina/22-000345-001/2021, and A/Ezo red fox/Hokkaido/1/2022) as well as the HPAI virus A/Feline/Korea/SNU-01/2023. Assays with vaccine strains were performed in enhanced BSL-2 facilities, whereas those involving the HPAI feline isolate were conducted under enhanced BSL-3 conditions. Serum samples were heat-inactivated, serially diluted two-fold starting at a 1:10 dilution, and incubated with the virus to allow neutralization. The serum–virus mixtures were then transferred onto 96-well plates seeded with MDCK cells. The MN titer was defined as the highest serum dilution that completely inhibited viral replication. Titers < 1:10 were regarded as negative and excluded from graphical presentation. Each serum sample was analyzed in at least three independent MN experiments.

### 2.5. Phylogenetic and Sequence Analysis of H5 Hemagglutinin (HA) Genes

The hemagglutinin (HA) gene sequences of H5 influenza viruses, including candidate vaccine viruses (CVVs) and representative field isolates, were obtained from the GISAID EpiFlu™ database. The HA sequence of A/feline/South Korea/SNU-01/2023 was obtained from our previous study. Sequence alignment and phylogenetic analysis were performed using MEGA version 7.0 (MEGA7). Phylogenetic analysis was conducted using the Maximum Likelihood (ML) method based on the Tamura–Nei substitution model. The initial tree for the heuristic search was generated automatically using the Neighbor-Joining and BioNJ algorithms applied to a matrix of pairwise distances estimated using the Maximum Composite Likelihood (MCL) approach. The topology with the highest log likelihood was selected as the final tree. The analysis included a total of 17 nucleotide sequences. All positions containing gaps and missing data were eliminated, resulting in a final dataset of 1692 positions. Branch support was assessed using bootstrap analysis with 1000 replicates. The phylogenetic tree was drawn to scale, with branch lengths representing the number of substitutions per site. Clade classification was determined according to the World Health Organization (WHO) H5 nomenclature system.

### 2.6. Statistical Analysis of Experimental Data

Statistical analyses were performed using GraphPad Prism™ software (version 9). Titers were log10-transformed prior to analysis. For comparisons of more than two groups, one-way analysis of variance followed by Tukey’s multiple comparisons test was applied.

For groups with two biological replicates (*n* = 2 ferrets), analyses were performed using mean titers from each animal to avoid pseudo-replication. Technical replicate measurements were used to confirm assay reproducibility and for graphical presentation. For the clade 1 Vietnam group, only one biological replicate (*n* = 1 ferret) was available; therefore, comparisons involving this group should be interpreted with caution.

*p* values are provided for descriptive reference only due to the limited number of animals per group.

## 3. Results

### 3.1. Generation of Ferret Antisera and Study Design

Ferret antisera were generated following immunization with four WHO-recommended CVVs: A/Vietnam/1194/2004 (Clade 1), A/Astrakhan/3212/2020 (Clade 2.3.4.4b), A/American wigeon/South Carolina/22-000345-001/2021 (Clade 2.3.4.4b), and A/Ezo red fox/Hokkaido/1/2022 (Clade 2.3.4.4b) (Figure 1). To ensure consistency with the licensed H5 vaccine currently used in Korea, which is formulated with alum as an adjuvant, the same adjuvant was applied for ferret immunization. Antisera collected two weeks after the final immunization were evaluated against homologous CVVs and a recent feline-derived HPAI H5N1 field isolate from Korea using HI and MN assays to assess cross-reactive antibody responses.

### 3.2. Cross-Reactive Antibody Responses Among CVVs

**Figure 2:**
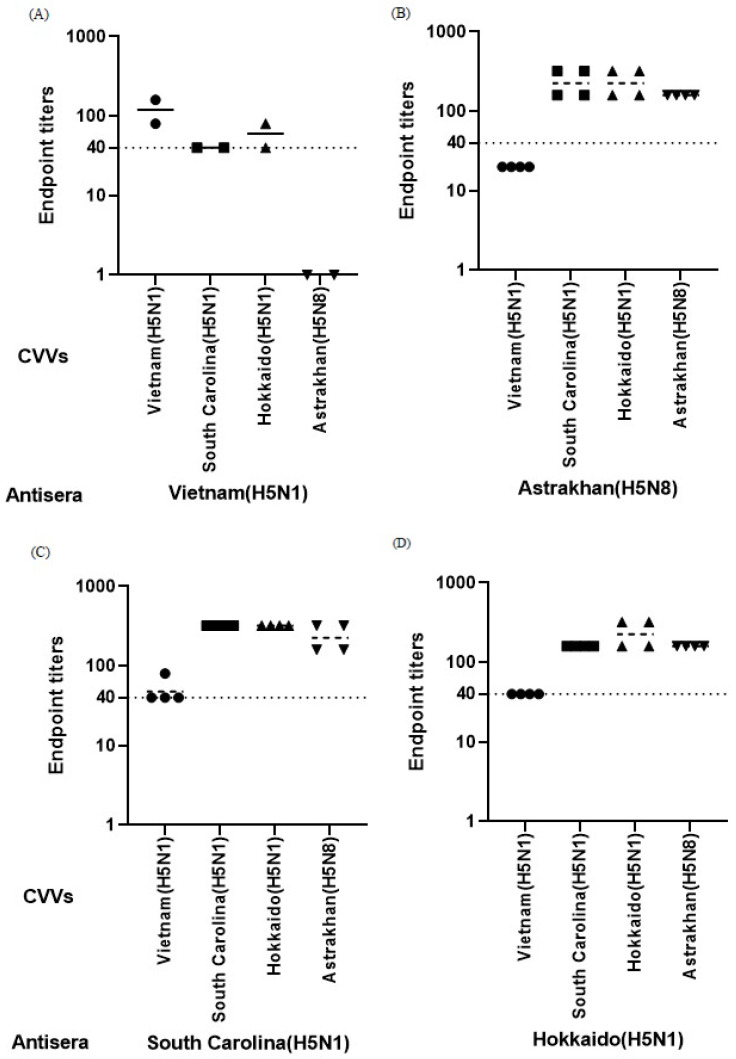
Hemagglutination inhibition (HI) titers of ferret antisera against homologous and heterologous candidate vaccine viruses (CVVs). HI titers were measured using antisera generated following immunization with WHO-recommended H5 CVVs. Ferrets were immunized with A/Vietnam/1194/2004 (**A**); A/Astrakhan/3212/2020 (**B**); A/American wigeon/South Carolina/22-000345-001/2021 (**C**); and A/Ezo red fox/Hokkaido/1/2022 (**D**). Each data point represents an individual measurement of serum obtained from immunized ferrets. Symbols indicate the CVVs used in the HI assay: circles, A/Vietnam/1194/2004; squares, A/American wigeon/South Carolina/22-000345-001/2021; upward triangles, A/Ezo red fox/Hokkaido/1/2022; downward triangles, A/Astrakhan/3212/2020. Horizontal bars indicate geometric mean titer (GMTs). The dotted line denotes the assay cutoff value (HI titer ≥ 40). Titers below the detection limit were assigned the lowest measurable value for graphical presentation. For panels (**B**–**D**) (*n* = 2 ferrets per group), similar patterns of cross-reactivity were observed across groups, whereas data in panel A were derived from a single ferret (*n* = 1). Given the limited number of animals per group, results are presented descriptively.

**Figure 3:**
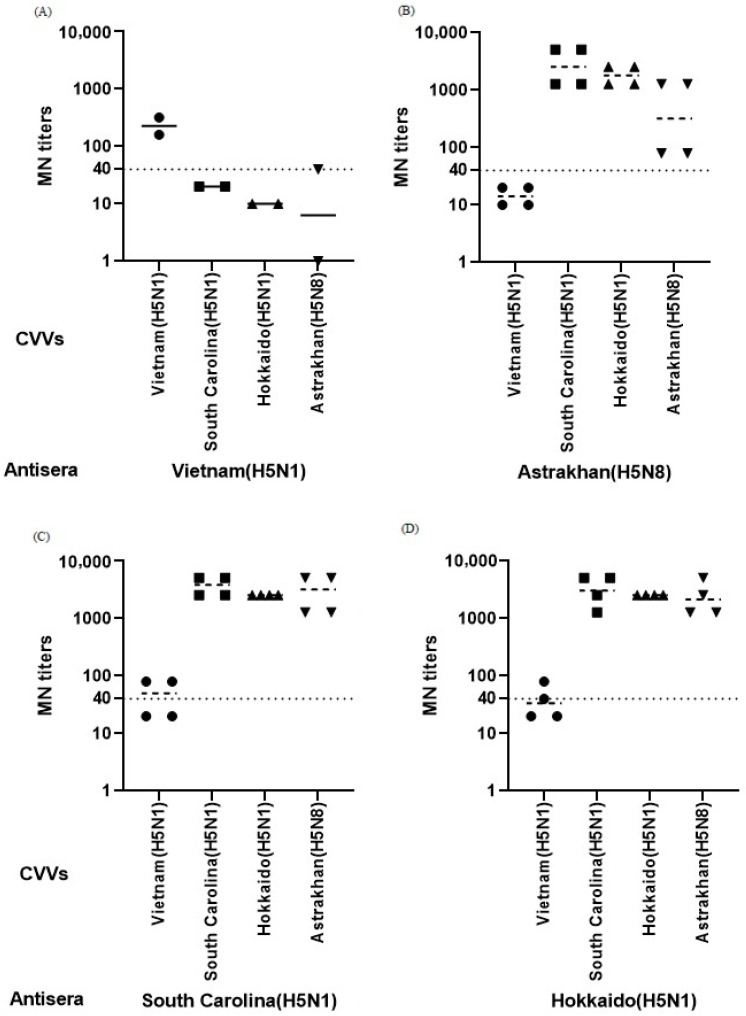
Microneutralization (MN) titers measured in the same ferret antisera against homologous and heterologous CVVs. MN titers were determined using antisera generated following immunization with WHO-recommended H5 CVVs as described in Figure 2. Ferrets were immunized with A/Vietnam/1194/2004 (**A**), A/Astrakhan/3212/2020 (**B**), A/American wigeon/South Carolina/22-000345-001/2021 (**C**), and A/Ezo red fox/Hokkaido/1/2022 (**D**). Each data point represents an individual measurement of serum obtained from immunized ferrets. Symbols indicate the CVVs used in the MN assay; circles, A/Vietnam/1194/2004; squares, A/American wigeon/South Carolina/22-000345-001/2021; upward triangles, A/Ezo red fox/Hokkaido/1/2022; downward triangles, A/Astrakhan/3212/2020. Horizontal bars indicate geometric mean titers (GMTs), and the dotted line represents the MN assay cutoff (MN titer ≥ 40). Titers below the detection limit were assigned the lowest measurable value for graphical presentation. Across panels, antisera induced by clade 2.3.4.4b CVVs showed cross-reactive antibody responses, whereas antisera induced by the Vietnam-derived clade 1 CVV exhibited limited reactivity. Variability among individual animals was observed, but overall patterns of neutralization were broadly similar across clade 2.3.4.4b groups. Given the limited number of animals per group, results are presented descriptively.

Ferret antisera generated following immunization with the clade 2.3.4.4b CVVs showed cross-reactive antibody responses against other clade 2.3.4.4b vaccine strains in both HI and MN assays (Figure 2 and Figure 3). Although variability in endpoint titers was observed among individual animals, similar patterns of cross-reactivity were observed across the two assay platforms. In contrast, antisera derived from ferrets immunized with the Vietnam-derived clade 1 CVV showed limited cross-reactivity against clade 2.3.4.4b CVVs. In the HI assay, heterologous titers were generally low and frequently at or below the assay cutoff, while MN titers were also lower compared with those elicited by clade 2.3.4.4b CVVs. These findings suggest antigenic differences between clade 1 and clade 2.3.4.4b H5 viruses at a descriptive level.

### 3.3. Comparative Neutralization Among Clade 2.3.4.4b CVVs

Among the clade 2.3.4.4b CVVs, antisera raised against the Astrakhan, South Carolina, and Hokkaido strains showed cross-reactive antibody responses against homologous and heterologous clade 2.3.4.4b viruses in MN assays (Figure 3B–D). Despite variability in endpoint titers among individual ferrets, the overall patterns of neutralization were broadly similar across the three clade 2.3.4.4b CVV groups. Panel-specific differences were observed in certain comparisons, but no consistent hierarchical pattern was identified among the three clade 2.3.4.4b CVVs. HI titers against clade 2.3.4.4b viruses were consistently lower than the corresponding MN titers and were distributed within a narrower dynamic range (Figure 2B–D). Across panels, antisera induced by clade 2.3.4.4b CVVs generally showed relatively higher HI and MN titers than those induced by the Vietnam-derived clade 1 CVV. Given the limited number of animals per group, these observations should be interpreted descriptively.

### 3.4. Cross-Neutralization of Feline-Origin H5N1 Virus

**Figure 4:**
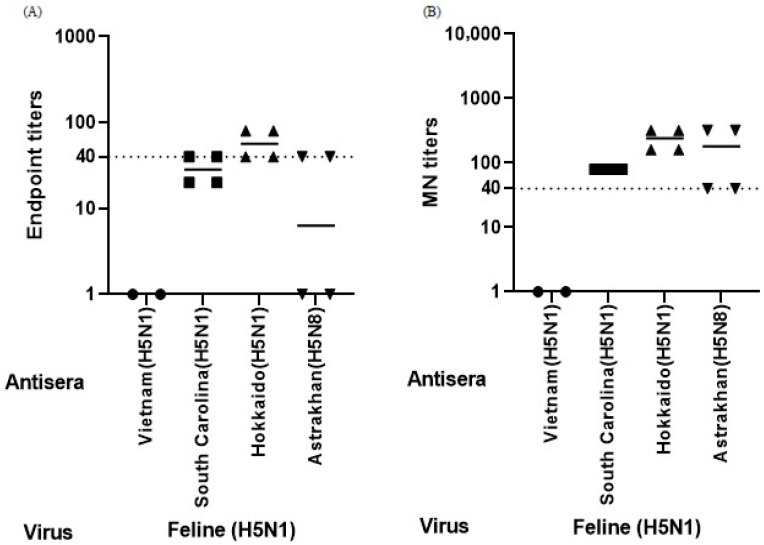
Hemagglutination inhibition (HI) and microneutralization (MN) titers of antisera against a feline-origin H5N1 virus. Cross-reactive antibody responses were evaluated against a feline-origin clade 2.3.4.4b H5N1 field isolate, A/Feline/Korea/SNU-01/2023, using HI (**A**) and MN (**B**) assays. Each data point represents an individual measurement of serum obtained from immunized ferrets. Symbols indicate the antisera group according to the CVV used for immunization: circles, A/Vietnam/1194/2004; squares, A/American wigeon/South Carolina/22-000345-001/2021; upward triangles, A/Ezo red fox/Hokkaido/1/2022; downward triangles, A/Astrakhan/3212/2020. Horizontal bars indicate geometric mean titers (GMTs), and dotted lines denote assay cutoff values (titer ≥ 40). Titers below the detection limit were assigned the lowest measurable value for graphical presentation. Antisera induced by clade 2.3.4.4b CVVs showed measurable cross-reactive responses, whereas antisera induced by the Vietnam-derived clade 1 CVV exhibited limited activity. Given the limited number of animals per group, results are presented descriptively.

To further evaluate the antigenic relevance of CVV-induced antisera against viruses at the human–animal interface, sera were tested against a feline-origin clade 2.3.4.4b H5N1 field isolate, A/Feline/Korea/SNU-01/2023, using both MN and HI assays (Figure 4). In MN assays, antisera raised against clade 2.3.4.4b CVVs showed relatively higher neutralizing titers against the feline-origin virus compared with antisera raised against the Vietnam-derived clade 1 CVV (Figure 4B), while no clear differences were observed among the clade 2.3.4.4b groups. In contrast, antisera from ferrets immunized with the Vietnam-derived clade 1 CVV exhibited limited neutralizing activity, remaining near the assay cutoff. In the HI assay, clade 2.3.4.4b CVV-induced antisera showed detectable activity against the feline-origin virus (Figure 4A). Compared with the Vietnam group, higher HI titers were observed for the South Carolina and Hokkaido groups, whereas the Astrakhan group showed similar levels to the Vietnam group. Collectively, these findings suggest that clade 2.3.4.4b CVVs are associated with cross-reactive antibody responses against a feline-origin H5N1 virus, whereas the clade 1 vaccine strain shows limited cross-reactivity.

### 3.5. Phylogenetic Relationships and Sequence Identity Analysis of H5 HA Genes

**Figure 5:**
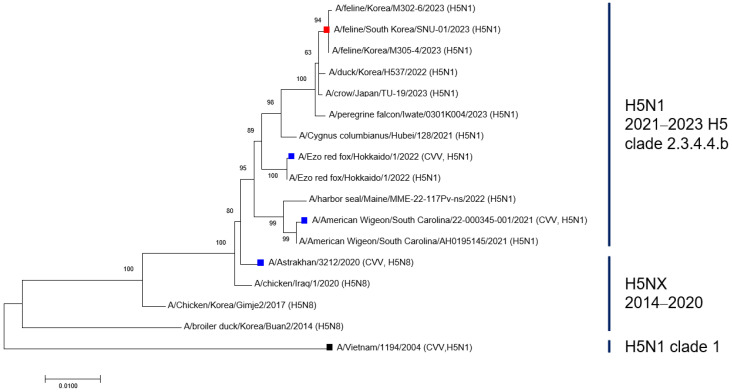
Molecular phylogenetic analysis of H5 hemagglutinin (HA) genes. The evolutionary history was inferred using the Maximum Likelihood method based on the Tamura-Nei model. The tree with the highest log likelihood is shown. Initial tree(s) for the heuristic search were generated automatically using the Neighbor-Joining and BioNJ algorithms applied to a matrix of pairwise distances estimated using the Maximum Composite Likelihood (MCL) approach, and the topology with the superior log likelihood value was selected. The analysis included 17 nucleotide sequences. All positions containing gaps and missing data were eliminated, resulting in a final dataset of 1692 positions. Bootstrap values (1000 replicates) are indicated at branch nodes. The tree is drawn to scale, with branch lengths measured in the number of substitutions per site. The feline-origin isolate A/feline/South Korea/SNU-01/2023 is indicated in red, WHO-recommended CVVs are highlighted in blue, and other reference strains are shown in black. Clade classifications are shown according to the WHO H5 nomenclature system.

Phylogenetic analysis of the hemagglutinin (HA) genes showed that A/feline/South Korea/SNU-01/2023 clustered within clade 2.3.4.4b, together with recently circulating H5N1 viruses and WHO-recommended candidate vaccine viruses (CVVs), including A/Ezo red fox/Hokkaido/1/2022, A/American wigeon/South Carolina/22-000345-001/2021, and A/Astrakhan/3212/2020. In contrast, the clade 1 vaccine strain A/Vietnam/1194/2004 formed a distinct and genetically distant lineage (Figure 5).

Consistent with phylogenetic clustering, amino acid sequence identity analysis revealed that A/feline/South Korea/SNU-01/2023 shared the highest identity with the clade 2.3.4.4b CVV A/Ezo red fox/Hokkaido/1/2022 (99.1%). High sequence identity was also observed with A/Astrakhan/3212/2020 (98.9%) and A/American wigeon/South Carolina/22-000345-001/2021 (98.4%). In contrast, sequence identity with the clade 1 CVV A/Vietnam/1194/2004 was substantially lower (91.7%) (Supplementary Table S1).

These results indicate that the feline-origin virus is genetically more closely related to clade 2.3.4.4b viruses than to the clade 1 lineage. This genetic relationship is consistent with the observed serological findings in this study, in which antisera raised against clade 2.3.4.4b CVVs showed cross-reactive antibody responses against the feline isolate, whereas antisera raised against the clade 1 CVV exhibited limited reactivity.

## 4. Discussion

Historically, newly emerging highly pathogenic avian influenza (HPAI) viruses have demonstrated the capacity for sporadic transmission to humans, often causing severe disease due to the lack of pre-existing immunity in human populations [22,23]. In the current pandemic era, marked by repeated zoonotic spillover and sustained global circulation of HPAI viruses, these observations highlight the importance of pandemic preparedness, particularly the development and stockpiling of vaccines capable of addressing antigenically evolving strains [24,25,26]. During the H5N1 outbreaks in Vietnam and Indonesia in the early 2000s, multiple vaccine formulations were evaluated, ultimately leading to the licensure of three H5 vaccines by the US Food and Drug Administration (FDA) [27,28,29].

The global spread of clade 2.3.4.4b H5 viruses across both avian and mammalian hosts has further increased concerns regarding zoonotic transmission and highlights vaccination as a critical countermeasure [30,31,32,33,34,35]. However, antigenic heterogeneity among influenza virus clades frequently limits cross-neutralization, underscoring the importance of selecting vaccine strains that are antigenically matched to currently circulating viruses [36,37,38]. Previous studies have shown that vaccines based on clade 1 and clade 2.1 viruses provide limited or inconsistent cross-protection against clade 2.3.4.4b viruses, largely reflecting genetic and antigenic divergence [39]. In contrast, vaccines formulated with more potent adjuvants, such as MF59 or AS03, have demonstrated broader antibody responses [40]. Nevertheless, despite the continued use of alum-adjuvanted clade 1 H5 vaccines in several preparedness programs, their cross-reactivity against contemporary clade 2.3.4.4b viruses, particularly mammalian spillover isolates-remains in sufficiently characterized.

Recent reports of clade 2.3.4.4b H5N1 infections in cats provide further evidence of ongoing avian-to-mammalian spillover at the human–animal interface. Although such infections have been documented in specific settings, phylogenetic and genomic analyses indicate that these viruses originate from avian H5N1 strains circulating along migratory bird flyways rather than sustained transmission within mammalian populations [16,41]. In line with these observations, the feline-origin virus A/Feline/South Korea/SNU-01/2023 analyzed in this study belongs to clade 2.3.4.4b and shows high genetic similarity to avian H5N1 viruses circulating in East Asia during the 2022–2023 winter season. Phylogenetic analysis further confirmed that this isolate clusters within clade 2.3.4.4b and is clearly separated from the clade 1 lineage. Consistently, amino acid sequence identity analysis demonstrated high similarity between the feline isolate and clade 2.3.4.4b CVVs (98.4–99.1%), whereas similarity with the clade 1 CVV was markedly lower (91.7%) (Table S1). These findings indicate that the feline isolate is genetically aligned with clade 2.3.4.4b viruses rather than with the clade 1 lineage.

This genetic relationship is reflected in the serological results. Antisera raised against clade 2.3.4.4b CVVs exhibited cross-reactive antibody responses against the feline isolate in microneutralization assays, whereas antisera raised against the clade 1 CVV showed limited reactivity. In hemagglutination inhibition (HI) assays, the differences were less pronounced and varied across panels, consistent with the lower sensitivity of HI compared with MN. Together with the homologous and heterologous comparisons among CVVs (Figure 2 and Figure 3), these findings support the presence of antigenic differences between the legacy clade 1 vaccine strain and currently circulating clade 2.3.4.4b viruses.

These findings differ from earlier reports describing partial cross-reactive antibody responses induced by alum-adjuvanted H5 vaccines against heterologous strains [42,43]. Such discrepancies may be explained by differences in antigenic distance, antigen dose, adjuvant formulation, vaccination schedule, and the use of animal versus human models. Although alum is a well-established adjuvant, its ability to broaden antibody responses may be limited when antigenic divergence is substantial. In contrast, oil-in-water adjuvants such as MF59 or AS03 have been shown to enhance both the magnitude and breadth of humoral responses [28,44,45]

Several limitations of this study should be considered. First, the number of animals per group was limited, and only one ferret was included in the clade 1 group, restricting statistical inference and necessitating cautious interpretation. Accordingly, this study was designed as an antigenic characterization and cross-immunogenicity assessment rather than a statistically powered comparative analysis. The results should therefore be interpreted descriptively, focusing on overall patterns of cross-reactivity rather than definitive quantitative comparisons. In addition, ferret sera were used instead of human sera. Nevertheless, ferrets are widely regarded as a relevant model for influenza antigenic characterization, and ferret antisera are routinely used by the World Health Organization and the Centers for Disease Control and Prevention in HI assays as a surrogate for human serology [46]. This study also focused on antibody-mediated responses assessed by HI and MN assays, whereas other immune components, including cellular and mucosal immunity, were not evaluated. Future studies incorporating larger cohorts, human sera, alternative adjuvants, and broader immunological endpoints will be important to further validate these findings.

Collectively, these findings emphasize the importance of evaluating vaccine candidates against viruses circulating at the human–animal interface and support the consideration of clade 2.3.4.4b-matched CVVs in pandemic preparedness and vaccine strain selection strategies [18,47]. Given the increasing detection of clade 2.3.4.4b H5N1 viruses in diverse mammalian hosts in Korea, these results further highlight the importance of selecting vaccine strains that are antigenically matched to currently circulating viruses within a One Health framework [17,48].

## 5. Conclusions

In conclusion, this study suggests that WHO-recommended clade 2.3.4.4b CVVs elicit cross-reactive antibody responses against a feline-origin clade 2.3.4.4b HPAI H5N1 isolate, whereas the clade 1 vaccine strain shows limited cross-reactivity [41]. Clade 2.3.4.4b CVVs generally induced higher antibody responses than the clade 1 vaccine strain, consistent with antigenic differences between legacy and contemporary H5 viruses. These findings are further supported by phylogenetic and sequence analyses demonstrating that the feline isolate is genetically more closely related to clade 2.3.4.4b viruses than to the clade 1 lineage. Given the ongoing global circulation of clade 2.3.4.4b H5N1 viruses across diverse avian and mammalian hosts, these results highlight the importance of vaccine strain selection strategies that consider viral evolution and cross-species transmission at the human–animal interface within a One Health framework. Collectively, our findings support the relevance of clade 2.3.4.4b-matched vaccine strains for pandemic preparedness. Future studies incorporating larger animal cohorts and human post-vaccination sera will be important to further validate the breadth and durability of cross-neutralizing responses. In addition, comparative evaluation of alternative adjuvant formulations and broader immunological endpoints, including cellular and mucosal immunity, will help define optimal vaccine strategies against antigenically evolving H5 viruses. Continued antigenic surveillance of mammalian spillover isolates will also be essential to guide timely updates of candidate vaccine viruses within a One Health framework.

## Data Availability

The data presented in this study are available within the article and Supplementary Materials. Additional data are available from the corresponding author upon reasonable request.

## Supplementary Materials

The following supporting information can be downloaded at: https://www.mdpi.com/article/10.3390/vaccines14040301/s1, Table S1: Amino acid sequence identity (%) of H5 hemagglutinin (HA) between the feline-origin isolate and representative candidate vaccine viruses.

## Abbreviations

The following abbreviations are used in this manuscript:

HPAI	Highly Pathogenic Avian Influenza
CVV	Candidate Vaccine Virus
WHO	World Health Organization
HI	Hemagglutination Inhibition
MN	Microneutralization
RBC	Red Blood Cell
RDE	Receptor-Destroying Enzyme
MDCK	Madin-Darby Canine Kidney
BSL	Biosafety Level
IACUC	Institutional Animal Care and Use Committee
ANOVA	Analysis of Variance
GMT	Geometric Mean Titer
CDC	Centers for Disease Control and Prevention
NIID	National Institute of Infectious Diseases
FDA	Food and Drug Administration
CBER	Center for Biologics Evaluation and Research
KDCA	Korea Disease Control and Prevention Agency
MF59	Squalene-based Oil-in-Water Adjuvant MF59
AS03	Adjuvant System 03 (Oil-in-Water Emulsion Adjuvant)
H5N1	Highly Pathogenic Avian Influenza A Virus subtype H5N1
H5	Hemagglutinin subtype 5
