# Supplementary material for: Evaluation of Cross-Immunogenicity of Ferret Antisera Following Immunization with H5N1 Vaccine Strains
Source: Vaccines (Basel). 2026 Mar 27;14(4):301. doi: 10.3390/vaccines14040301 (PMC13120575; doi:10.3390/vaccines14040301)
Supplement: Supplementary file 1 [file vaccines-14-00301-s001.zip › vaccines-4224977-supplementary.pdf]

	A/Vietnam/1194/2004	A/American Wigeon/South Carolina/22-000345-001/2021	A/Ezo red fox/Hokkaido/1/2022	A/Astrakhan/3212/2020
**A/feline/South Korea/SNU-01/2023**	91.7	98.4	99.1	98.9

Table S1. Amino acid sequence identity (%) of H5 hemagglutinin (HA) between the feline-origin isolate and representative candidate vaccine viruses. Amino acid sequence identity (%) of H5 hemagglutinin (HA) between A/feline/South Korea/SNU-01/2023 and representative WHO-recommended candidate vaccine viruses (CVVs), including A/Vietnam/1194/2004, A/American wigeon/South Carolina/22-000345-001/2021, A/Ezo red fox/Hokkaido/1/2022, and A/Astrakhan/3212/2020. Sequence identity values were calculated based on aligned HA amino acid sequences using MEGA7 and provide a quantitative measure of genetic relatedness among the analyzed strains, consistent with the phylogenetic analysis.
